# Research landscape and thematic evolution of venlafaxine and paroxetine discontinuation: a hybrid bibliometric analysis integrating web of science and scopus (2000–2025)

**DOI:** 10.3389/fmed.2026.1937438

**Published:** 2026-10-27

**Authors:** Zhishou Liu, Jing Huang, Ying Tang, Yonghui Huang

**Affiliations:** 1 Department of Pharmacy, Da Zhu Hospital, Dazhou, China; 2 Department of Gastroenterology, Da Zhu Hospital, Dazhou, China

**Keywords:** antidepressant withdrawal, bibliometrics, discontinuation syndrome, paroxetine, venlafaxine

## Abstract

**Background:**

Due to their shorter half-lives, paroxetine and venlafaxine often induce symptoms of discontinuation. There is still a lack of a thorough macroscopic analysis of the historical development and structure of this particular area.

**Methods:**

The literature on this topic was collected from Web of Science and Scopus (2000–2025) and analyzed using bibliometric analysis. Descriptive and collaboration analyses utilized the merged dataset, whereas citation networks were constructed exclusively using Web of Science data. All data synthesis and visualizations were done in R using the bibliometrix, igraph, and ggplot2 packages, applying giant component extraction and the Fruchterman-Reingold force-directed layout for network optimization.

**Results:**

A total of 903 documents were included. The number of annual publications reached its maximum around 2007 and since then has remained stable. The publications were primarily concentrated in North America and Europe, with the United States and the United Kingdom being the leading contributors. The clinical manifestations reported as “anxiety” and “withdrawal” were still basic themes identified in the results of the keyword analysis and thematic analysis. Interestingly, the research emphasis is now moving away from case reports of early phenomenology to “tapering” and “pharmacokinetics” (underlying strategies). Co-citation networks revealed a persistent theoretical impact from the pioneering psychopharmacologists and early specialized studies.

**Conclusions:**

Venlafaxine and paroxetine withdrawal studies have evolved from clinical understanding to mechanistic investigation and protocol creation. Embedding some of the tapering ideas within a general treatment paradigm may offer a potential pathway to support clinical outcomes.

## 1 Introduction

Selective serotonin reuptake inhibitors (SSRIs) and serotonin-norepinephrine reuptake inhibitors (SNRIs) are commonly used for treating depression and anxiety disorders. Although there is general consensus on their clinical effectiveness, clinical management of treatment cessation remains a challenge (1). Among these drugs, paroxetine and venlafaxine stand outas the most problematic agents during dose reduction. Unlike other antidepressants, these two drugs are characterized by exceptionally short elimination half-lives and an absence of long-acting active metabolites. Combined with their potent serotonin reuptake inhibition profiles, these pharmacokinetic properties make abrupt discontinuation of use, or even a lowering of the dosage, often trigger severe physical and psychological responses (2). A drug-specific bibliometric analysis of these two agents is therefore warranted to isolate the research dynamics of the most challenging clinical scenarios from broader antidepressant studies.

These clinical reactions are traditionally known as “antidepressant discontinuation syndrome” but are increasingly referred to as withdrawal syndrome. As articulated by scholars such as G.A. Fava, the use of the term “withdrawal” provides a broader clinical framework, emphasizing that symptoms can occur during dose reduction, may persist for extended periods, and represent distinct clinical phenomena rather than relapse or disease recurrence (3). Importantly, this terminology focuses on physiological neuroadaptation without implying psychological addiction or a substance dependence disorder. Common symptoms include dizziness, sensations of electric shock, gastrointestinal disturbances, and rebound anxiety (2). These symptoms can sometimes complicate tapering, and result in longer and more unplanned use of medication (4). In addition, treatment of drug withdrawal in susceptible groups (pregnant women) presents another clinical dilemma (5). Hence, a thorough understanding and proper management of the risks associated with paroxetine and venlafaxine withdrawal have become a major area of interest in psychopharmacology.

To address these issues, a considerable amount of literature, including case reports, randomized controlled trials, and clinical guidelines, has been published in the last few decades (2). The current knowledge base, however, seems to be very fragmented (6). Although systematic reviews are useful in synthesizing clinical data, they generally have limited methodological tools to quantitatively assess the field's evolution on a macro-scale level (7). While a recent comprehensive bibliometric study by Akça successfully mapped the broader landscape of SSRI and SNRI withdrawal research over three decades (8), there remains a need for a highly granular analysis focusing exclusively on the most high-risk agents. The specific evolutionary trajectories, distinct collaborative networks, and foundational literature uniquely addressing venlafaxine and paroxetine withdrawal require further delineation.

The current study aims to complement existing literature by systematically reviewing the literature on venlafaxine and paroxetine discontinuation and analyzing it bibliometrically. In this study, two databases, Web of Science and Scopus, were used to reduce the retrieval bias and to build an integrated database (9). We aim to visualize and map publication patterns, scientific collaboration networks, conceptual structures and citation histories using advanced computational tools. Finally, this macro-level analysis could help clinicians and researchers identify historical patterns and trends and envision future developments in creating safer antidepressant tapering protocols (10).

## 2 Materials and methods

### 2.1 Data sources and search strategy

Data retrieval was conducted using two major international citation databases, namely Web of Science Core Collection (WoS) and Scopus. A dual database search strategy was used to cover the literature. The search query included words that would capture documents pertaining to venlafaxine and paroxetine discontinuation or withdrawal. The search string used in the topic fields (TS in WoS and TITLE-ABS-KEY in Scopus) was: (“venlafaxine” OR “effexor” OR “paroxetine” OR “paxil” OR “seroxat”) AND (“discontinuation syndrome” OR “withdrawal syndrome*” OR “withdrawal symptom*” OR “discontinuation symptom*” OR “brain zap*” OR “antidepressant withdrawal” OR “tapering”). Standalone terms such as “discontinuation” or “withdrawal” were purposely excluded to prevent the massive retrieval of unrelated clinical trials where these terms merely denoted “patient withdrawal from the study” or “treatment discontinuation due to unrelated adverse events.” It was decided that the retrieval period would be from 1 January 2000 until 31 December 2025. The inclusion criteria was limited to original articles and review papers published in English, to ensure academic rigor. During this preliminary filtering, 86 non-English records (approximately 3.7% of initial hits, primarily published in German, French, Spanish, and Chinese) were excluded. The primary search was conducted on February 15, 2026.

### 2.2 Data extraction and preprocessing

The retrieved records were then exported with complete metadata, such as cited references (Plain text format for WoS and BibTeX format for Scopus). All data processing was performed in the R software environment. The datasets were synthesized by combining the metadata of both databases and systematically removing exact duplicates by combining Digital Object Identifiers (DOI) and article titles. To address the broad nature of the initial search query and eliminate false-positive results (e.g., studies primarily focusing on opioid withdrawal, neuropathic pain, or neonatal abstinence where target antidepressants were only incidentally mentioned), a rigorous manual title and abstract screening was implemented. A total of 410 irrelevant records were excluded. The detailed study selection flow is illustrated in Supplementary Figure S1. Following this screening, the refined dataset of 903 records was retained for subsequent analyses. To clarify the application of datasets: the merged and deduplicated WoS-Scopus dataset was utilized for all descriptive analyses, publication trends, spatial mapping, and collaboration network construction. Conversely, because of severe incompatibilities in the raw cited reference string formats between Scopus and WoS (which prevents accurate matrix calculation and risks double-counting citations), all co-citation networks (authors, references, and sources) and the historical direct citation analyses were conducted exclusively using the standardized WoS dataset. In addition, an approach with text preprocessing and keyword normalization using regular expressions was applied to the author keywords before the conceptual analysis. Prior to collaboration analyses, author and institution names were manually reviewed and disambiguated (e.g., unifying different abbreviations for the same university) to ensure accurate productivity metrics. General terms such as “antidepressant,” “antidepressants,” “SSRI,” “SSRIS,” “serotonin,” “treatment,” “therapy,” “review,” “depression,” “paroxetine,” and “venlafaxine” were excluded as stopwords. Synonyms and plural forms were systematically harmonized: “withdrawal symptoms” and “withdrawal syndrome” were merged into “WITHDRAWAL”; “discontinuation synd.” and “discontinuation syndrome” into “DISCONTINUATION”; and “anxiety disorder” and “anxiety disorders” into “ANXIETY.” Additionally, punctuation variations (e.g., hyphens in “obsessive-compulsive disorder”) were standardized into unified terms. For visualizations, explicit thresholds were applied: the top 20 terms were retained for the tree map, the top 35 interconnected nodes for the co-occurrence network, and terms with an occurrence threshold of *N* ≥ 5 for the trend topics timeline.

### 2.3 Bibliometric and statistical analysis

Bibliometric indicators such as publication frequencies, cumulative growth and the Hirsch index (H-index) were calculated using the bibliometrix package (v4.1.3) within the R environment (v4.3.0). For network visualizations, nodes were typically restricted to the top 30–50 entities based on occurrence frequency. During the network analysis, two kinds of adjacency matrices were produced: a collaboration matrix and a co-citation matrix. The igraph package was used to remove isolated nodes and small fragmented components from the networks (the so-called “hairball effect” in complex network visualizations), and to obtain the giant component of the networks (the largest connected subgraph). This procedure retained approximately 80% to 85% of core interconnected entities, deliberately omitting highly isolated clinical trial groups to accurately capture mainstream collaborative relationships. For network visualizations, inclusion thresholds were set to the top 30–40 nodes based on connection strength. The Fruchterman–Reingold force-directed algorithm was used to optimize the network layouts. Strategic quadrants were built to analyse the thematic map using the Walktrap clustering algorithm based on network topological features; the relevance degree (centrality) was calculated with the PageRank algorithm, and the development degree (density) was calculated with local transitivity. The ggplot2 package was used to perform all visualizations, and the ggrepel algorithm to calculate the optimal repulsive forces to prevent overlapping between node texts. There was no hypothesis testing, as bibliometric parameters are by and large descriptive parameters.

## 3 Results

### 3.1 General characteristics, publication trends, and source dynamics

The retrieved literature is summarized in the initial overview in Supplementary Table S1. After screening, a total of 903 documents published from the period 2000–2025 were included in the final analysis and represent a comprehensive data set to evaluate research on venlafaxine and paroxetine discontinuation.

The scientific production in this period is shown in Figure 1A. The number of publications showed a rapid rise with a record high of 65 articles in 2006–2007. The annual production then slowly waned and over the last 10 years has remained at about 20–30 articles per year. This inverted *U*-shaped pattern may have a close relationship with the history of clinical awareness of antidepressant withdrawal. This increase in publications in the 2000s may reflect the first reports and cautions regarding discontinuation reactions, which led to many observations and case reports. The stabilization that followed could be associated with maturation in the basic recognition of the syndrome, coinciding with a gradual shift in research focus towards specific management strategies and pharmacological mechanisms.

**Figure 1:**
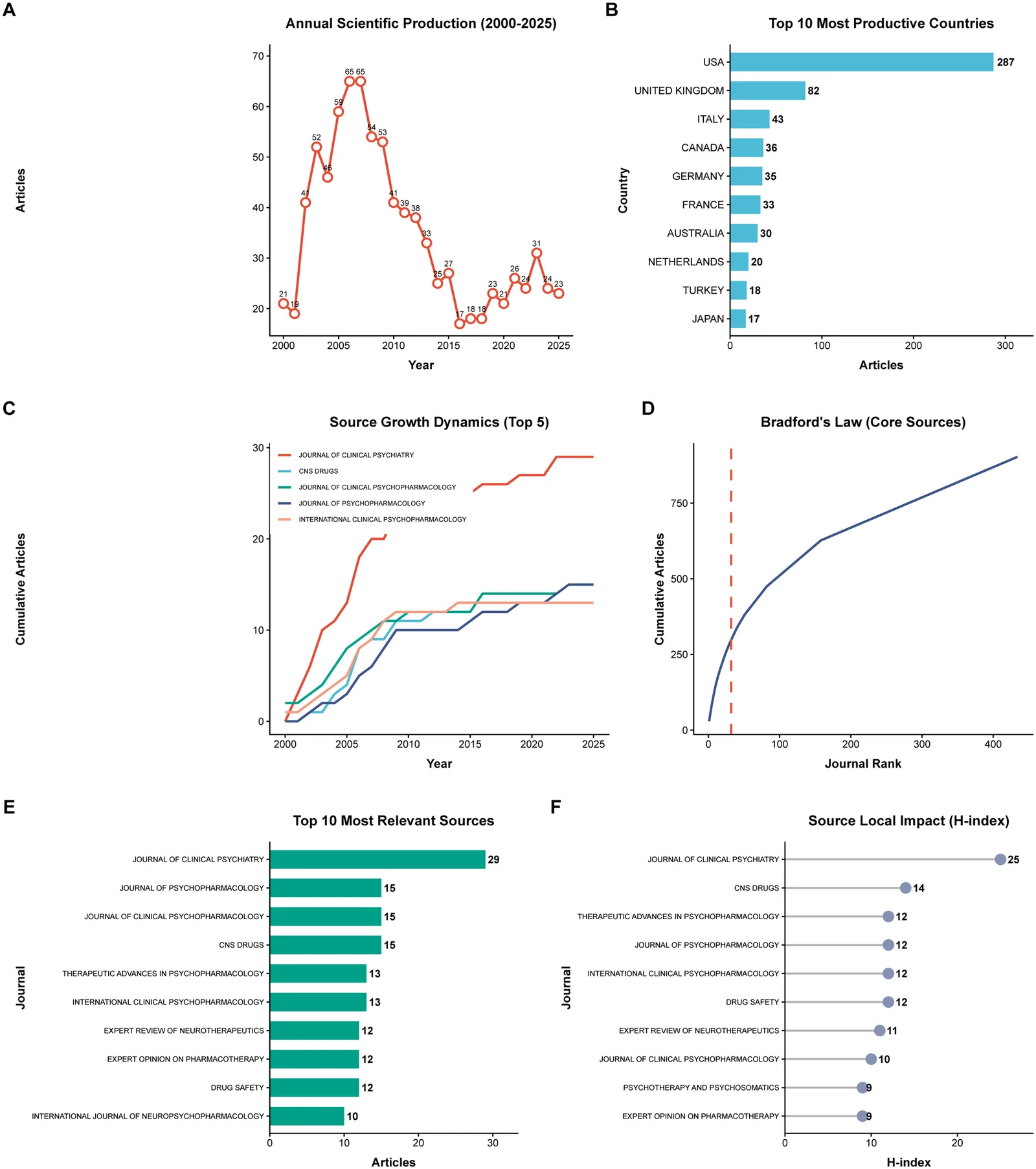
General characteristics and publication trends of the retrieved literature on venlafaxine and paroxetine discontinuation (2000–2025, *N* = 903). **(A)** Annual scientific production over the 25-year period. **(B)** Top 10 most productive countries based on total publications. **(C)** Cumulative source growth dynamics of the top five journals. **(D)** Bradford law analysis identifying the core sources in Zone 1. **(E)** Top 10 most relevant sources based on publication count. **(F)** Source local impact evaluated by the Hirsch index (H-index) within the analyzed dataset.

In terms of geographical distribution, the United States was the most productive country with 287 articles, followed by the United Kingdom with 82 articles and Italy with 43 articles (Figure 1B). This distribution shows that Western countries have been the main sources in the creation of the knowledge base for this particular research field.

To further analyze the dynamics of publication sources, core journals that disseminated these findings were identified. Bradford law analysis (Figure 1D) identified that there was a very compact core zone (Zone 1) consisting of a small number of journals publishing a high percentage of the relevant literature. Of these core sources, Journal of Clinical Psychiatry was found to be the most relevant journal with the highest number of publications (29 articles) (Figure 1E). In addition, the cumulative growth trend (Figure 1C) and local impact assessment (Figure 1F) consistently revealed that Journal of Clinical Psychiatry had the highest local H-index (25) in this set of journals, followed by CNS Drugs. These results suggest that mainstream psychiatric and clinical psychopharmacology journals are the primary venues where discussion takes place regarding venlafaxine and paroxetine discontinuation.

### 3.2 Distribution and collaboration networks of countries, institutions, and authors

Supplementary Table S2 extends the geographical results above and shows the most productive and highly cited entities in this field. Collaboration networks and production dynamics were then visualized at macro, meso and micro levels to explore further the interactive relationship among these entities.

On the macro level, Figure 2B shows the percentage of single country publications compared to multiple country publications for the highest publishing countries. The USA had a large number of publications, but the majority of these were single country publications, and there was a relatively low percentage of international collaboration. Nevertheless, as shown by the country collaboration network (Figure 2A), research activities in the world are highly interconnected, with Western countries (such as the USA, UK, Germany and Italy) as the main collaborative hubs, and some emerging nodes starting to integrate into the network.

**Figure 2:**
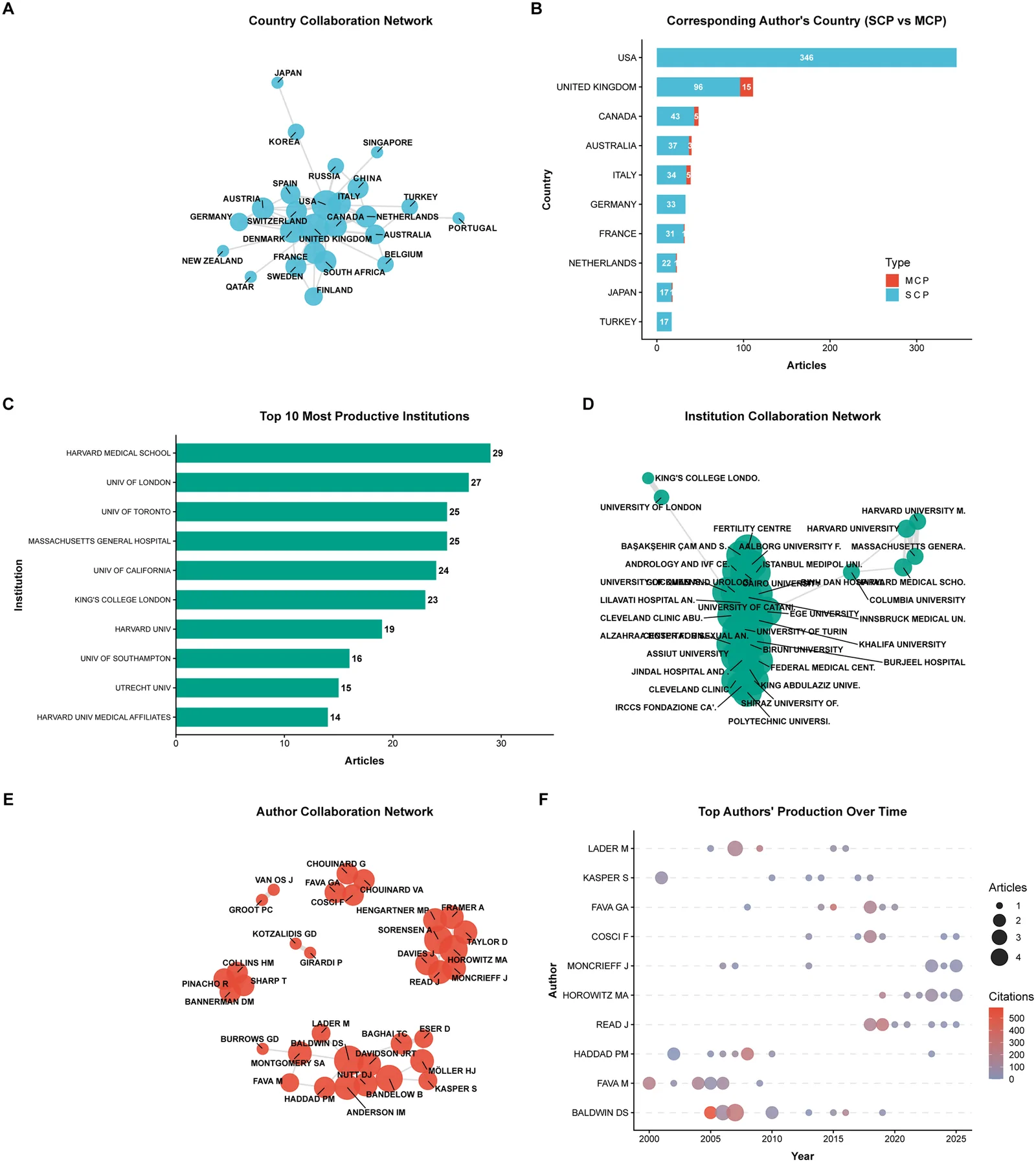
Distribution and collaboration networks of countries, institutions, and authors. **(A)** Collaboration network among countries. **(B)** Corresponding authors country, categorized by single country publications (SCP, domestic research) and multiple country publications (MCP, international collaboration). **(C)** Top 10 most productive institutions. **(D)** Collaboration network among institutions. **(E)** Collaboration network among authors. **(F)** Production over time for the top authors. In network diagrams **(A,D,E)**, node size reflects the degree of collaboration, and edge thickness indicates collaboration strength; isolated nodes were omitted using the Fruchterman-Reingold layout. In panel **(F)**, bubble size indicates publication frequency, and color depth reflects total citations (TC).

At the meso level, the top 10 most productive institutions were mostly North American institutions, with the University of Toronto, the University of California and Harvard Medical School at the top (Figure 2C). The institution collaboration network (Figure 2D), which captures the largest connected component of international partnerships, shows a rich network of diverse international universities and medical centers that are actively collaborating. The fact that not all the top institutions from Figure 2C are found in the core of this particular cohesive sub-network suggests that some of the top-producing institutions might tend to work in isolation or in smaller, very specialized local networks. The giant component that can be visualized, by contrast, likely represents clinical or observational consortiums, which are broader and multi-center.

Author contribution and interaction were explored at the micro level. As shown in Supplementary Table S2, scientists with high publications like Baldwin DS (11), Haddad PM (12), and Fava M (13) also have high scientific impact, in terms of total citations and h-indices. Figure 2E, the author collaboration network, shows that there are tightly knitted research groups, indicating that studies on antidepressant discontinuation are often research group-based rather than individual researcher-based. Specifically, distinct collaborative clusters are evident among core researchers, such as the psychosomatic research network [e.g., Fava GA (14) and Cosci F (15)], the contemporary safe tapering community [e.g., Horowitz MA (10), Taylor D (16), and Read J (17)], and established psychopharmacology pioneers [e.g., Baldwin DS (11), Haddad PM (18), and Montgomery SA (19)]. In addition, the temporal production chart (Figure 2F) has been implemented to show the life cycle of the core authors. During the 2000s, there were a number of highly active pioneers, such as Baldwin DS and Fava M, who had a darker bubble, indicating that they had more total citations. It is a common bibliometric phenomenon, as older basic research studies will have a longer period of time to accrue citations. At the same time, there has been a recent upsurge of research and interest by others, like Horowitz MA (20) and Moncrieff J (21), suggesting a generational change, or a renewed focus on certain aspects of the discontinuation syndrome in modern-day psychiatry.

### 3.3 Conceptual structure, hotspots, and thematic evolution

Author keywords were then analyzed to define the core concepts and reveal the evolution trend of research hotspots after the main contributors in this area were identified. To increase the accuracy of the semantic analysis, background search terms (e.g., antidepressants, venlafaxine, paroxetine) were filtered out and synonyms were clustered together before mapping.

Figures 3A,B show the static conceptual framework. The tree map (Figure 3A) shows the frequency of core keywords in a hierarchical manner, with the most frequent keywords on the left and the least frequent ones on the right, and “discontinuation,” “anxiety,” “withdrawal,” and “pregnancy” being the top four most frequent clinical aspects. The co-occurrence network (Figure 3B) also provides additional details on how these concepts interact. The nodes are grouped around the core themes and represent specific clinical manifestations (e.g., panic disorder, insomnia) and pharmacological management strategies (e.g., tapering, pharmacotherapy), with each grouping being closely concentrated around the core themes.

**Figure 3:**
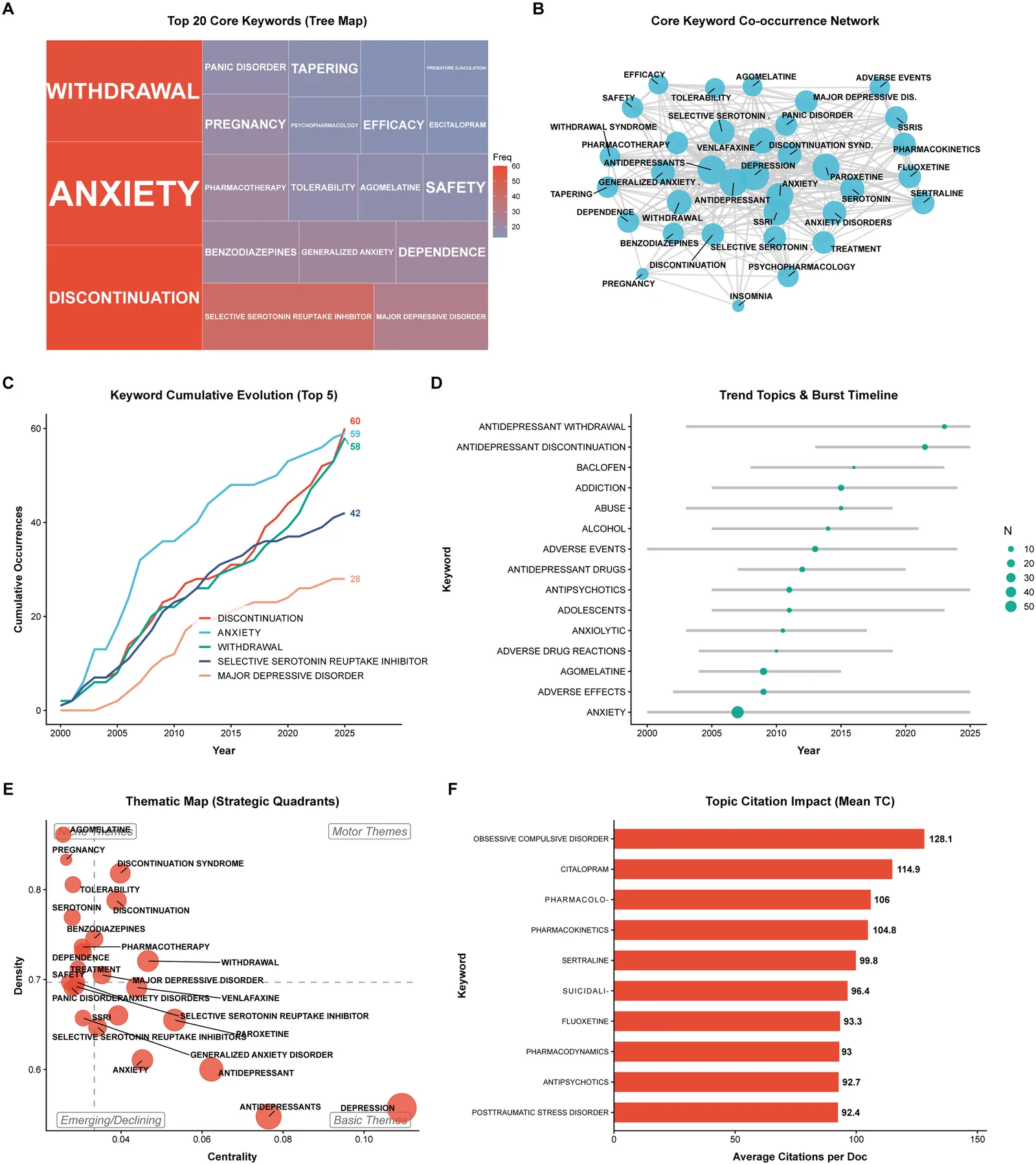
Conceptual structure, hotspots, and thematic evolution based on author keywords. **(A)** Tree map of the top 20 normalized core author keywords. **(B)** Keyword co-occurrence network, where node size represents occurrence frequency and edge thickness denotes co-occurrence strength. **(C)** Cumulative evolution trajectories of the top five keywords over time. **(D)** Timeline of trend topics, displaying the lifespan of high-frequency keywords. **(E)** Thematic map distributing keywords into four strategic quadrants based on relevance degree (centrality) and development degree (density). **(F)** Topic citation impact based on the mean total citations (Mean TC) per document for core topics.

A cumulative occurrence and a trend topics timeline were plotted to depict the temporal evolution of these concepts. The top five keywords are shown to have a marked upward trajectory over the last 25 years in Figure 3C. Importantly, “withdrawal” and “discontinuation” exhibit pronounced upward trajectories in Figure 3C, but with a distinct temporal inflection. Historically, “discontinuation” was the predominant term established in the early 2000s, largely adhering to official consensus frameworks aimed at differentiating antidepressant cessation from substance abuse disorders. However, from approximately 2015 onwards, the term “withdrawal” gained significant momentum, accelerating at a steeper rate and emerging as a prominent trend topic in recent years (Figure 3D). This chronological shift reflects an evolving nosological debate, wherein academic literature has increasingly adopted “withdrawal” to align with patient-reported severity and physiological receptor neuroadaptation. With the burst timeline (Figure 3D), changes in the topics can be observed in a fine-grained way. When dividing the 25-year study period into three distinct historical phases, a clear thematic transition emerges: Early Phase (2000–2007): Research was primarily characterized by broad phenomenological recognition and acute clinical descriptions (e.g., “drug therapy,” “panic disorder”), stimulated by early regulatory alerts regarding discontinuation emergent events. Intermediate Phase (2008–2015): The literature expanded toward high-risk clinical contexts and comparative tolerability, with prominent attention directed toward maternal and fetal safety (“pregnancy”) and adverse drug reactions. Contemporary Phase (2016–2025): A paradigm shift is evident, as recent trend topics are dominated by “pharmacology,” “pharmacokinetics,” and actionable management strategies such as “tapering.” This trajectory suggests that academic focus has progressed from merely cataloging discontinuation symptoms toward deciphering neurobiological mechanisms and developing structured cessation protocols. Whereas general “drug therapy” and “panic disorder” were the predominant subjects in earlier literature, in recent years topics like “sleep,” “pharmacology,” and “pharmacokinetics” have become trend topics. This change may reflect an evolving research interest, transitioning from early broad phenomenological reporting toward specialized investigations into clinical symptomatology and underlying pharmacokinetic parameters.

Lastly, strategic quadrants and citation impacts were analyzed to assess the developmental status of these topics. The keywords are classified using the thematic map (Figure 3E), which was created based on centrality and density. In the basic themes quadrant, “withdrawal” stands out prominently, highlighting its fundamental role within this domain. At the same time, topics such as “tapering” and “addiction” are placed close to or inside of the motor and niche themes, suggesting they could serve as catalysts for further specialized research. As for academic influence, Figure 3F demonstrates that articles focusing on obsessive compulsive disorder received the highest average citations (128.1 citations per document), followed by specific comparator antidepressants such as citalopram (114.9), sertraline (99.8), and fluoxetine (93.3), as well as mechanistic domains including pharmacology and pharmacokinetics. This phenomenon suggests that comparative studies—frequently comparing the short half-life of venlafaxine or paroxetine with longer acting alternatives such as fluoxetine—are classical study designs in the examination of discontinuation risks, and thus consistently receive higher citation rates.

### 3.4 Knowledge base, intellectual structure, and citation analysis

A historical direct citation network was created (Figure 4A) to gain insight into the historical heritage of the hotspots mentioned above. Interestingly, although the main data set dates from 2000 to 2025, the root nodes of this network go back to the 1990s (e.g., Coupland NJ, 1996; Rosenbaum JF, 1998). This distribution may indicate that the basic clinical observations regarding venlafaxine and paroxetine withdrawal were made early on in the drugs’ history, before the wave of research that occurred after the year 2000.

**Figure 4:**
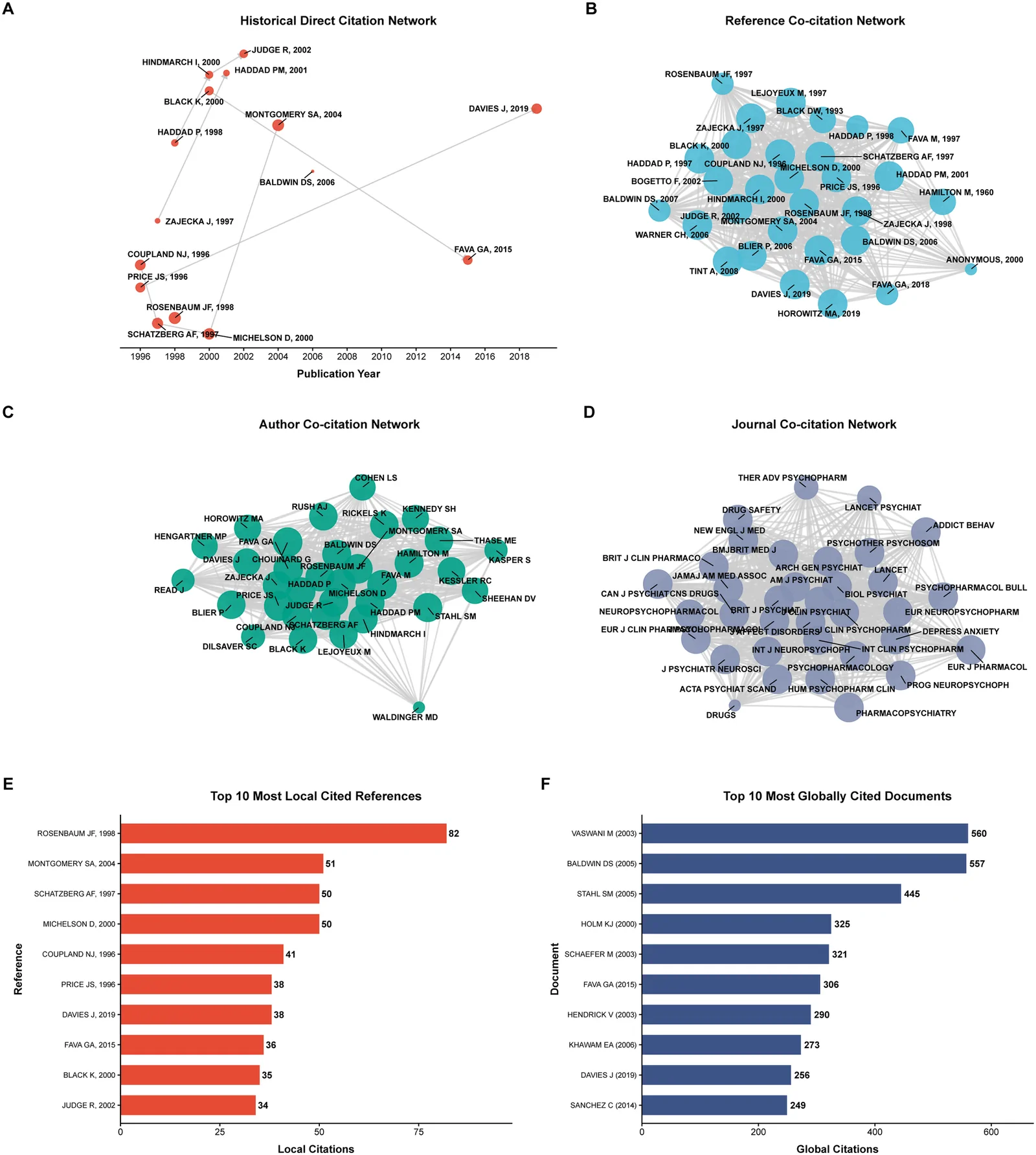
Intellectual structure, knowledge base, and citation analysis. **(A)** Historical direct citation network displaying early seminal documents, with horizontal position indicating publication year (restricted to integers). **(B)** Reference co-citation network. **(C)** Author co-citation network, excluding anonymous records. **(D)** Journal co-citation network. In network plots **(B–D)**, node size denotes co-citation frequency and links represent co-citation relationships. **(E)** Top 10 most locally cited references within this specific dataset. **(F)** Top 10 most globally cited documents across Web of Science and Scopus.

Co-citation analyses were then used to further map the underlying knowledge base. The reference co-citation network (Figure 4B) shows how the seminal articles are grouped together and are cited together, thus providing the theoretical underpinning of this field. Likewise, the author co-citation network (Figure 4C) reveals key authors including Fava M, Baldwin DS and Rosenbaum JF. Their prominence is consistent with the productivity metrics shown earlier, suggesting their ongoing theoretical influence on academic discourse. In addition, the journal co-citation network (Figure 4D) shows that the co-citation pattern is very dense, with co-citations concentrated in major psychiatric and psychopharmacological journals, including the American Journal of Psychiatry and the Journal of Clinical Psychiatry, indicating a very focused disciplinary origin.

The local and global citations were systematically compared to assess the specific impact of pivotal documents. The most highly cited references in this specific dataset are identified in Figure 4E, with early specialized studies, such as Rosenbaum JF (1998) and Schatzberg AF (1997)/Montgomery SA (2004), being most highly cited. These documents may be the most direct and frequently referenced references for researchers who are particularly interested in discontinuation syndromes. On the other hand, the top 10 most cited documents worldwide in the entire database are listed in Figure 4F and Supplementary Table S3. Global citations of foundational review papers like Vaswani M (2003) and clinical guidelines such as Baldwin DS (2005) were substantial, reflecting broad academic visibility rather than direct evidence of therapeutic superiority. This contrast may suggest that the documents with the greatest macro-level impact—broader clinical guidelines or a large-scale systematic review that includes the specific drug risks—differ from the documents that are most important in this niche, which are the early specific phenomenological reports.

## 4 Discussion

Venlafaxine and paroxetine, both highly effective treatments for depressive and anxiety disorders, are also being studied for their tendency to cause very severe withdrawal symptoms, because of their short half-lives (2). This study offers a thorough bibliometric mapping of the research field around this clinical phenomenon over the last 25 years, showing that scientific work on this has mostly been descriptive of the phenomenon in the beginning and is currently veering in the direction of specific tapering strategies, pharmacological mechanisms and general consensus building.

When compared to the broader bibliometric analysis of SSRI/SNRI withdrawal by Akça (8), which demonstrated a continuous linear growth in overall literature, our drug-specific mapping reveals a distinct narrative. The early stabilization of publications specific to venlafaxine and paroxetine suggests that the clinical discourse for these highest-risk, short-half-life agents matured more rapidly than for the broader antidepressant class. Furthermore, while Akça identified general thematic trends, our focused analysis isolated hyper-specific clinical challenges—such as maternal risks (“pregnancy”) and the pressing need for tailored “pharmacokinetic” tapering protocols—that disproportionately affect patients discontinuing these two specific drugs.

A clear trajectory exists in the publication trend that was identified in this study. In contrast to the steady linear increase observed in general psychiatric research publications, the publications on venlafaxine and paroxetine discontinuation reached a maximum around 2007 and have since remained fairly constant. This is in line with the changes in clinical awareness over time. Observational studies were likely prompted by the major regulatory warnings of the early 2000s, after early case reports in the late 1990s (22). The subsequent plateau might reflect a stabilization in the primary clinical identification of the syndrome. Thematic evolution analysis, however, shows that there has been a distinct thematic transition in the research focus. The words “pharmacology” and “pharmacokinetics” are among the latest hot spots as shown by the trend topics timeline. This change from mere descriptive studies to mechanistic explorations is similar to the emphasis of some recent reviews in the neurobiological field, which focus on the need to understand the down-regulation of receptors during the quick withdrawal of drugs, and not simply list clinical symptoms (23).

Moreover, the keyword co-occurrence network and thematic map identified “anxiety”, “withdrawal” and “pregnancy” as the main research areas. Of particular interest is the consistent co-occurrence of the terms “withdrawal” and “discontinuation” revealed in our temporal mapping. While “discontinuation syndrome” was favored for nearly two decades to prevent patient stigmatization, the post-2015 acceleration of “withdrawal” identified in our keyword dynamics indicates a conceptual paradigm shift. As articulated by contemporary critics, the term “withdrawal” is increasingly utilized to validate persistent clinical phenomena and recognize that gradual hyperbolic neuroadaptation is required during dose reduction, bridging the gap between clinical terminology and patient lived experiences. Although “discontinuation syndrome” has been used traditionally to distinguish these symptoms from substance abuse, many independent researchers are now calling for the use of “withdrawal” based on broader clinical arguments. As noted by G.A. Fava, this terminology better captures the potential for persistent symptoms and helps differentiate new withdrawal phenomena from underlying disease relapse, without necessarily implying classical substance dependence (2). Our results reflect this duality of terminology, suggesting that consensus in psychiatric terminology is still an open question. Further, the citation analysis showed a contrast between the local and global impacts. Within this niche, early and very specific studies provide the theoretical basis, while comprehensive clinical guidelines have a broader influence in the world (22). This implies that embedding specific drug risks in broader treatment plans is a highly effective means of dissemination for research results to medical practitioners beyond the narrow confines of researchers (6).

Methodologically, two databases (Web of Science and Scopus) were combined to reduce the coverage gap of using only one database (9). Although there may be risks of loss of citation data during a merge because of incompatible metadata structures, this study utilized robust data cleaning and priority deduplication algorithms aimed at preserving the integrity of core citation networks. As a result, the historical direct citations and co-citations networks and maps exhibit strong intellectual structures and little degradation of the data. Furthermore, the use of a high threshold for the filter of high-frequency terms proved to be an effective method for removing irrelevant outlier terms from the thematic evaluations, so that only the terms of interest (relative to the pharmacological phenomena targeted) were retained.

These bibliometric trajectories offer critical implications for both clinical practice and future research agendas. For clinical practice, the persistent prominence of withdrawal phenomena and comparative pharmacology underscores that antidepressant cessation can be protracted and challenging, particularly for paroxetine and venlafaxine due to their short half-lives. Frontline clinicians, particularly in primary care, must distinguish new withdrawal symptoms from underlying illness relapse, avoiding premature re-escalation of dosages. Furthermore, the emerging focus on pharmacokinetics supports the growing clinical consensus advocating for hyperbolic tapering—reducing doses in progressively smaller increments to accommodate non-linear serotonin transporter receptor occupancy—rather than rapid linear reductions over 2–4 weeks. For future research, while scientific interest in tapering protocols has expanded, there remains a critical dearth of randomized controlled trials comparing different discontinuation regimens. Future investigations must prioritize prospective comparative trials evaluating specific tapering modalities (such as liquid formulations or tapering strips) and elucidate the neurobiological correlates governing individual susceptibility to withdrawal.

Importantly, a crucial methodological distinction must be emphasized: while our bibliometric mapping illustrates an accelerating scientific interest in tapering and pharmacokinetic regimens, this study does not assess, compare, or endorse the clinical efficacy, tolerability, or safety of any specific tapering protocol. Clinicians must not interpret keyword prominence or citation metrics as empirical therapeutic evidence; clinical tapering decisions must remain guided by dedicated randomized trials and formal clinical practice guidelines.

Despite those methodological considerations, there are two main limitations of this study. First, non-English publications and gray literature were not included. This linguistic restriction inevitably favored Anglophone countries (such as the United States and the United Kingdom) and internationalized European academic centers. Consequently, clinical observations and localized tapering protocols published in native-language journals across non-Anglophone regions in Asia, Latin America, and Europe were underrepresented in our global collaboration mapping (9). Second, potential search-term bias may exist, and the decision to exclude broader terms like “discontinuation” alone to avoid noise might have inadvertently missed some relevant studies. Third, citation counts are inherently affected by citation-age bias, heavily favoring older publications. Fourth, the methodological choice to filter networks to their giant components inevitably excluded smaller, isolated research communities. Finally, bibliometric analyses are based on quantitative information that is susceptible to historical bias. Specifically, citation-based metrics (such as the mean citations used to evaluate topic impact) are inherently confounded by publication age, heavily favoring older foundational concepts over newly emerging themes (24). Future systematic reviews and meta-analyses are therefore needed to examine the clinical effectiveness of the various tapering approaches suggested to date in these research hotspots (25).

## 5 Conclusion

This bibliometric analysis provides a macro-level overview of the literature on venlafaxine and paroxetine discontinuation spanning 25 years. The findings reveal a thematic evolution from early phenomenological case reports toward mechanistic investigations and the development of specific tapering strategies. Core clinical domains, including withdrawal symptoms, rebound anxiety, and pregnancy-related risks, remain prominent research hotspots. Furthermore, citation structures demonstrate that while early specialized reports established the field's foundation, comprehensive clinical guidelines currently command the broadest global impact. Ultimately, this study maps the historical trajectories and structural networks of this domain, highlighting key knowledge gaps to inform future directions in psychopharmacological research. Crucially, while this study maps the expanding scientific literature surrounding tapering protocols, it does not assess which specific discontinuation strategy is clinically superior or safer; evaluating therapeutic efficacy requires rigorous prospective clinical trials and systematic reviews.

## Data Availability

The original contributions presented in the study are included in the article/Supplementary Material, further inquiries can be directed to the corresponding author.
