# Supplementary material for: Research landscape and thematic evolution of venlafaxine and paroxetine discontinuation: a hybrid bibliometric analysis integrating web of science and scopus (2000–2025)
Source: Front Med (Lausanne). 2026 Oct 27;13:1937438. doi: 10.3389/fmed.2026.1937438 (PMC13748361; doi:10.3389/fmed.2026.1937438)
Supplement: Supplementary file 2 [file Supplementaryfile1.docx]

**Supplementary Figure S1. PRISMA-style flow diagram of the study selection process.** The flowchart details the identification, screening, and inclusion phases of the literature retrieval. A manual screening step was implemented to exclude false-positive records (e.g., studies primarily related to opioid withdrawal or neuropathic pain) where the target antidepressants were only incidentally mentioned, providing a refined dataset for the final bibliometric analysis.
