# Supplementary material for: Research landscape and thematic evolution of venlafaxine and paroxetine discontinuation: a hybrid bibliometric analysis integrating web of science and scopus (2000–2025)
Source: Front Med (Lausanne). 2026 Oct 27;13:1937438. doi: 10.3389/fmed.2026.1937438 (PMC13748361; doi:10.3389/fmed.2026.1937438)
Supplement: Supplementary file 3 [file Table1.docx]

Table S1. Main characteristics of the retrieved literature (2000–2025)

| **Description** | **Results** |
| --- | --- |
| **MAIN INFORMATION ABOUT DATA** |  |
| Timespan | 2000 – 2025 |
| Sources (Journals, Books, etc.) | 434 |
| Documents | 903 |
| Annual Growth Rate (%) | 0.36 |
| Document Average Age (Years) | 15.3 |
| Average citations per document | 38.55 |
| Average citations per year per document | 2.62 |
| References | 7,126 |
| **DOCUMENT TYPES** |  |
| Article | 469 |
| Review | 394 |
| Letter | 11 |
| Editorial material | 10 |
| Article; proceedings paper | 10 |
| Meeting abstract | 7 |
| Other document types | 2 |
| **DOCUMENT CONTENTS** |  |
| Keywords Plus (ID) | 6,259 |
| Author's Keywords (DE) | 1,311 |
| **AUTHORS AND COLLABORATION** |  |
| Total Authors | 2,718 |
| Author Appearances | 3,178 |
| Authors of single-authored documents | 162 |
| Single-authored documents | 195 |
| Documents per Author | 0.33 |
| Co-Authors per Document | 3.52 |
| International co-authorships (%) | 5.43 |

Note: Data were obtained from the combined and deduplicated Web of Science Core Collection and Scopus databases after rigorous text-filtering and screening.

Table S2. Top 10 most productive and impactful entities (Authors, Countries, and Institutions)

| **Category** | **Entity** | **Total Publications (TP)** | **Total Citations (TC)** | **H-index** |
| --- | --- | --- | --- | --- |
| **Author** | BALDWIN DS | 15 | 1,275 | 12 |
|  | FAVA M | 10 | 593 | 10 |
|  | READ J | 9 | 571 | 7 |
|  | HADDAD PM | 9 | 508 | 8 |
|  | HOROWITZ MA | 8 | 453 | 8 |
|  | MONCRIEFF J | 8 | 218 | 7 |
|  | MONTGOMERY SA | 7 | 745 | 7 |
|  | FAVA GA | 7 | 686 | 7 |
|  | LADER M | 7 | 534 | 7 |
|  | COSCI F | 7 | 281 | 7 |
| **Country** | USA | 287 | - | - |
|  | UNITED KINGDOM | 82 | - | - |
|  | ITALY | 43 | - | - |
|  | CANADA | 36 | - | - |
|  | GERMANY | 35 | - | - |
|  | FRANCE | 33 | - | - |
|  | AUSTRALIA | 30 | - | - |
|  | NETHERLANDS | 20 | - | - |
|  | TURKEY | 18 | - | - |
|  | JAPAN | 17 | - | - |
| **Institution** | HARVARD MEDICAL SCHOOL | 29 | - | - |
|  | UNIVERSITY OF LONDON | 27 | - | - |
|  | MASSACHUSETTS GENERAL HOSPITAL | 25 | - | - |
|  | UNIVERSITY OF TORONTO | 25 | - | - |
|  | UNIVERSITY OF CALIFORNIA | 24 | - | - |
|  | KING'S COLLEGE LONDON | 23 | - | - |
|  | HARVARD UNIVERSITY | 19 | - | - |
|  | UNIVERSITY OF SOUTHAMPTON | 16 | - | - |
|  | UTRECHT UNIVERSITY | 15 | - | - |
|  | HARVARD UNIVERSITY MEDICAL AFFILIATES | 14 | - | - |

Note: TP = Total Publications; TC = Total Citations; H-index = Hirsch index calculated based on the analyzed dataset. "NA" author records resulting from anonymous publications were removed to ensure author metric accuracy. Citation metrics for countries and institutions were omitted as the primary focus is on macro-level productivity.

Table S3. Top 10 most globally cited documents in the field (2000–2025)

| **Rank** | **Document (First Author, Year, Source)** | **Title** | **DOI** | **Total Citations (TC)** | **TC per Year** |
| --- | --- | --- | --- | --- | --- |
| 1 | VASWANI M, 2003, PROG NEURO-PSYCHOPHARMACOL | Role of selective serotonin reuptake inhibitors in psychiatric disorders: A comprehensive review | 10.1016/S0278-5846(02)00338-X | 560 | 23.33 |
| 2 | BALDWIN DS, 2005, J PSYCHOPHARMACOL | Evidence-based guidelines for the pharmacological treatment of anxiety disorders: Recommendations from the British Association for Psychopharmacology | 10.1177/0269881105059253 | 557 | 25.32 |
| 3 | STAHL SM, 2005, CNS SPECTRUMS | SNRIs: The pharmacology, clinical efficacy, and tolerability in comparison with other classes of antidepressants | 10.1017/S1092852900019726 | 445 | 20.23 |
| 4 | HOLM KJ, 2000, DRUGS | Zolpidem: An update of its pharmacology, therapeutic efficacy and tolerability in the treatment of insomnia | 10.2165/00003495-200059040-00014 | 325 | 12.04 |
| 5 | SCHAEFER M, 2003, HEPATOLOGY | Adherence and mental side effects during hepatitis C treatment with interferon alfa and ribavirin in psychiatric risk groups | 10.1053/jhep.2003.50031 | 321 | 13.38 |
| 6 | FAVA GA, 2015, PSYCHOTHER PSYCHOSOM | Withdrawal symptoms after selective serotonin reuptake inhibitor discontinuation: A systematic review | 10.1159/000370338 | 306 | 25.50 |
| 7 | HENDRICK V, 2003, AM J PSYCHIATRY | Placental passage of antidepressant medications | 10.1176/appi.ajp.160.5.993 | 290 | 12.08 |
| 8 | KHAWAM EA, 2006, CLEVELAND CLIN J MED | Side effects of antidepressants: An overview | 10.3949/ccjm.73.4.351 | 273 | 13.00 |
| 9 | DAVIES J, 2019, ADDICT BEHAV | A systematic review into the incidence, severity and duration of antidepressant withdrawal effects: Are guidelines evidence-based? | 10.1016/j.addbeh.2018.08.027 | 256 | 32.00 |
| 10 | SANCHEZ C, 2014, INT CLIN PSYCHOPHARMACOL | A comparative review of escitalopram, paroxetine, and sertraline: Are they all alike? | 10.1097/YIC.0000000000000023 | 249 | 19.15 |

Note: TC = Total Citations across Web of Science and Scopus databases; DOI = Digital Object Identifier; TC per Year was calculated based on the timespan up to 2026. Highly cited articles primarily reflect foundational pharmacological frameworks, clinical treatment guidelines, and systematic evaluations of antidepressant cessation phenomena.
